# Retraction Note: Ocean dynamic equations with the real gravity

**DOI:** 10.1038/s41598-022-10846-0

**Published:** 2022-04-26

**Authors:** Peter C. Chu

**Affiliations:** Department of Oceanography, Naval Postgraduate School, Monterey, CA USA

Retraction of: *Scientific Reports* https://doi.org/10.1038/s41598-021-82882-1, published online: 05 February 2021

The Editors have retracted this Article.

After publication of this study concerns were raised that the study is based on a flawed premise that a horizontal component of gravity is neglected in oceanographic calculations. In practice, this component can be taken to be zero, because the errors associated with this neglect are smaller than the error of assuming the resting ocean surface appears locally level, as shown by Chang and Wolfe^1^. This is further expanded upon in Stewart and McWilliams^2^, who also show that even in a model formulated in absolute spherical coordinates, the horizontal component of gravity has a negligible impact on ocean circulation.

The Editors no longer stand by the conclusions of this Article.

Peter Chu disagrees with this retraction.
